# Unconventional Obturator Artery Nutrient Branch: Image of an Anatomical Variation

**DOI:** 10.3390/diagnostics12082019

**Published:** 2022-08-21

**Authors:** Benjamin L. Bosse, Victoria J. Palacios, Dustin W. Dutcher, Emily J. Etter, Peter C. Lim, Caroline A. Cobine, Gillian L. Moritz

**Affiliations:** 1 Department of Physiology & Cell Biology, Reno School of Medicine, University of Nevada, Reno, NV 89557, USA; 2 Center of Hope—Gynecologic Oncology, Pelvic and Robotic Surgery, Reno, NV 89511, USA; 3 Office for Community Faculty, Reno School of Medicine, University of Nevada, Reno, NV 89557, USA

**Keywords:** obturator artery, internal iliac artery, aberrant, nutrient artery, pelvic vasculature, anatomical variations

## Abstract

Variations in vascular anatomy are of great concern to surgeons, as proper identification of aberrant arteries can reduce the risk of iatrogenic injury and improve patient outcomes. Several studies have highlighted the irregular branching pattern of pelvic arteries, with a recent focus on the obturator artery (OA). The OA has an inconstant origin from the internal iliac artery, external iliac artery, or inferior epigastric artery. Within the pelvis, the OA can give off muscular branches and nutrient vessels to the ilium and pubis. Though occasionally described in text, few resources employ images of human donors that depict branches arising from the OAs. Out of the 34 hemisected pelves studied, we identified 1 individual with a substantial nutrient vessel branching unilaterally from the OA. Herein, we present the first image of this unconventional nutrient artery. This vessel should be highlighted given that its size and course make it particularly vulnerable during intrapelvic surgeries such as pelvic lymph node dissection or in procedures requiring arterial embolization of the OA.

**Figure 1:**
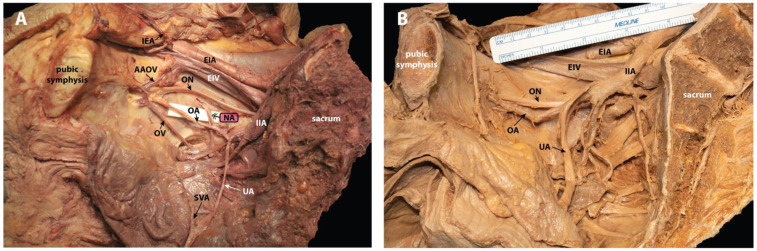
Pelvic hemisection of an unconventional nutrient artery (NA) and a typical vascular pattern in phenol-fixed human donors. Donor bodies were dissected as part of the gross anatomy curriculum for medical students at the University of Nevada, Reno School of Medicine. (**A**) Aberrant NA of the obturator artery (OA) in a right pelvic hemisection of a 65-year-old Caucasian female with a listed cause of death of acute respiratory disease, chronic obstructive pulmonary disease, and pneumonia. The bisected sacrum is seen on the right and the pubic symphysis is on the left of the image. Anterior to the sacrum, the right common iliac artery (not visible here) branches into the EIA and IIA. The EIA is seen superior to the EIV. The IIA continues and bifurcates into anterior and posterior divisions. The first branch off the anterior division is the UA, and it is reflected medially. The SVA is seen branching off the UA to supply the urinary bladder. The OA can be seen traveling from the anterior division of the IIA to the obturator foramen with an aberrant NA (double arrowhead) coursing inferior to the arcuate line and through an unnamed foramen. This NA is not depicted in other studies of the OA, nor was it found in our examination of 34 other hemisected pelves. The inferior vesical, vaginal, middle rectal, internal pudendal, and inferior gluteal arteries are not labeled in this image. The patient had a history of hysterectomy, and as a result, the uterine artery was obliterated. Seen exiting through the obturator foramen with the OA is the OV, with an AAOV, as well as the ON. The posterior division branches of the IIA are not visible in this image. (**B**) Typical vasculature seen in a right pelvic hemisection. In this donor, the OA originates from the IIA and courses to the obturator foramen without giving off any substantial nutrient branches. The obturator artery (OA) is typically a branch of the anterior division of the internal iliac artery (IIA), but the occurrence of aberrant OAs is relatively common [1,2,3,4,5,6,7,8,9,10,11]. Accordingly, the origin of the OA is well studied, and can be classified using various schemes [12,13]. Sañudo et al. (2011) categorized OA variations into six different types, the most common being the OA arising from the internal iliac artery (IIA; 35.5%) and from the inferior epigastric artery (IEA; 22.5%). The OA of the human donor shown in panel A arises from the anterior trunk of the internal iliac artery. In our sample of 34 pelvic hemisections, we predominantly found an OA origin from IIA (76.5%) with relatively fewer origins from the IEA (14.7%). While categorizations of the origin of the OA are well established, photographic depictions of the muscular and nutrient branches arising from the OA are limited. In the human donor case presented here, the OA arises from the anterior division of the IIA and proceeds through the obturator canal leaving the pelvis. Prior to exiting the pelvis, however, it gives off a significant arterial branch that dives anterolaterally and passes through an unnamed foramen towards the arcuate line of the ilium (panel A). While the internal aspect of the ilium is supplied by nutrient arteries arising from the obturator or iliolumbar arteries, these are typically small-diameter vessels that are unremarkable in the course of dissection and are of little clinical significance (panel B). Furthermore, the details of these branches are often limited to written descriptions or outdated illustrations in the published literature [14,15,16]. The NA seen above is substantial, and images of any similar vessels are absent in the literature. The artery depicted here provides an important reference image, and it is of particular note clinically for a range of specialties, including gynecology, radiology, orthopedics, and urology [17]. NA = Nutrient Artery; AAOV = Aberrant Accessory Obturator Vein; IEA = Inferior Epigastric Artery; EIA = External Iliac Artery; EIV = External Iliac Vein; IIA = Internal Iliac Artery; OA = Obturator Artery; ON = Obturator Nerve; OV = Obturator Vein; SVA = Superior Vesical Artery; UA = Umbilical Artery [18].
